# Revisiting the Quality of the Scottish Record Linkage System.

**DOI:** 10.23889/ijpds.v7i3.1940

**Published:** 2022-08-25

**Authors:** David Clark, John Reid, Dave Bailey, Suhail Iqbal, Heather Clydesdale, Ken Humphreys, Jade Carruthers, Lena Henderson, Martin Reid, Adebusola Ramsay

**Affiliations:** 1 Public Health Scotland; 2 National Records of Scotland

## Objectives

To evaluate the quality of linkage carried out by operators in the Trusted Third Parties (TTPs) in Public Health Scotland (PHS) and National Records of Scotland (NRS).

To compare results with a PHS automated production system.

To produce individual reports to feedback results to operators, informing areas for improvement.

## Approach

A test dataset (n=274,479) containing names, dates of birth, sex and postcode, was derived from hospital episodes and additional synthetic records added. For a proportion of records, some variables were perturbed. The true reference index number was retained to compare against the indexes returned by operators from matching to the population spine.

A run of the Medical Record Linkage (MRL) system (used to routinely link the centrally held hospitalisations, cancer and death registrations over the last 25 years) was executed without clerical review, to provide a benchmark for the TTPs.

Recall, precision and F-measures were calculated to compare results.

## Results

There were 5 reports produced, including the MRL run. NRS contributed a single run, and PHS-TTP offered 3. Quality overall was excellent, with some variation dependent on operator, methods, and spine used. The MRL run was ranked 3rd overall for F-measure, with overall precision of 99.69% and 97.36% recall. Where data had been unperturbed precision was 99.92% with 99.90% recall. The best ranked run was carried out by a member of PHS TTP, with 99.72% precision (99.93% unperturbed) and 98.77% recall (99.94% unperturbed). NRS results showed precision of 99.56% (99.91% unperturbed) and 96.71% recall (99.80% unperturbed).

Individual reports were produced and tailored to highlight the types of perturbations where each operator performed strongly or otherwise, encouraging operators to learn and adapt processes in future.

## Conclusion

Data Linkage in Scotland has evolved over 5 decades, more recently with the introduction of TTPs as key support services for research projects. However, little evaluation of the quality of linkage has been conducted. The results demonstrated by this exercise provide re-assurance to researchers that linkage quality remains high

